# Application of Table Tennis Ball Trajectory and Rotation-Oriented Prediction Algorithm Using Artificial Intelligence

**DOI:** 10.3389/fnbot.2022.820028

**Published:** 2022-05-11

**Authors:** Qiang Liu, Hairong Ding

**Affiliations:** Shanghai Polytechnic University, Shanghai, China

**Keywords:** artificial intelligence, machine learning, track recognition of table tennis, human motion recognition, support vector machines algorithm

## Abstract

The present work aims to accelerate sports development in China and promote technological innovation in the artificial intelligence (AI) field. After analyzing the application and development of AI, it is introduced into sports and applied to table tennis competitions and training. The principle of the trajectory prediction of the table tennis ball (TTB) based on AI is briefly introduced. It is found that the difficulty of predicting TTB trajectories lies in rotation measurement. Accordingly, the rotation and trajectory of TTB are predicted using some AI algorithms. Specifically, a TTB detection algorithm is designed based on the Feature Fusion Network (FFN). For feature exaction, the cross-layer connection network is used to strengthen the learning ability of convolutional neural networks (CNNs) and streamline network parameters to improve the network detection response. The experimental results demonstrate that the trained CNN can reach a detection accuracy of over 98%, with a detection response within 5.3 ms, meeting the requirements of the robot vision system of the table tennis robot. By comparison, the traditional Color Segmentation Algorithm has advantages in detection response, with unsatisfactory detection accuracy, especially against TTB's color changes. Thus, the algorithm reported here can immediately hit the ball with high accuracy. The research content provides a reference for applying AI to TTB trajectory and rotation prediction and has significant value in popularizing table tennis.

## Introduction

Modern technologies like artificial intelligence (AI) have become the forefront of research with continuous science and technological advancement. AI is also known as machine intelligence and computer intelligence. As the name implies, this technology aims to intellectualize machines or computers like humans (Hu, 2020).

Artificial intelligence has seen two significant elements: summarization and logical deduction, regarded as the connectionism approach and the symbolism approach, respectively (Riguzzi et al., 2019; Park and Hainaut, 2020). Human beings process audio-visual signals based on cortical neural networks without thinking. This learning method is called the connectionism approach. Accordingly, connectionism scholars do not investigate the deep-seated learning process in their research but obtain the final result through machine learning algorithms learning a large number of data and methods autonomously. Human's mathematical derivation and proving theorem are based on solid subjective consciousness and axiomatic system, requiring conscious thinking and symbolic calculus. This learning method is called the symbolism approach (Lin et al., 2020). Symbolism scholars tend to design formulas to solve problems based on this definition. They investigate the deep-seated calculation process and obtain the final result through the complete process of machine learning (Al-Mukhtar and Al-Yaseen, 2020; Gomez-Gonzalez et al., 2020). The connectionism approach and symbolism approach are most common in the current AI field. Other algorithms are based on the extension of the two methods in all directions. A table tennis robot (TTR) is an advanced intelligent robot (IR) with comprehensive solid quality, consisting of vision, decision-making, and control systems. These robots can respond to external stimuli, thereby realizing man-machine confrontation in various scenes. TTR can assist professional player training well (Likitha, 2021) and is significant in popularizing table tennis sports. Additionally, IR is also of great significance (Carreras et al., 2020).

The innovation of this paper lies in the following aspects. First, the IR is used in table tennis training, and the table tennis ball (TTB) trajectory is predicted and calculated based on a deep learning algorithm. Secondly, a machine learning algorithm is proposed to identify motion states and the rotation and orbit of TTB. In short, AI technology is applied to table tennis training and competition to predict and determine the TTB trajectory accurately.

## Relevant Theories and Methods

### The Application of AI in the Field of Sports

With the continuous development of AI technology, IRs have been multi-functionalized and highly intellectualized. In particular, IRs have been extensively applied in many sports, including table tennis, badminton, basketball, and football. Among those sports IRs, TTR has some unique and delicate features worthy of in-depth exploration. For example, the TTB is very light and moves extremely fast, up to 5–20 m/s. Therefore, the TTR must be sensitive, accurate, and robust to lend itself well to train or play with professional players (Steiner et al., 2020). Thus, target identification and trajectory prediction of the table tennis ball (TTB) are incredibly complicated, becoming the key points and difficulties in the current research field (Zhang et al., 2020). The TTR-based TTB recognition depends on a vision system to analyze the hitting actions of the table tennis player and predict the real-time position and motion state of TTB. Thus, the TTR's vision system must have the ability to predict trajectory and plan actions for the target to ensure detection accuracy and real-time motion recognition (Zhao et al., 2021).

This paper studies the TTR from three aspects (Forghani, 2020): vision, decision-making, and control systems. Among them, the vision system distinguishes TTR from other sports IRs. The three procedures are interdependent and work collaboratively, each with different target detection and trajectory prediction tasks. First, the vision system of the TTR is an upgraded version of general machine learning vision systems and the eye to detect and track the real-time position and dynamic states of TTB. The vision system has a solid ability to track high-speed moving objects (Li, 2021; Zhang, 2021). Second, the decision-making system is the instruction-distributor of the three systems. After receiving the information transmitted by the vision system, the decision-making system needs to respond accordingly to predict the TTB trajectory (Gomis-Fons et al., 2021). Moreover, the vision system is also responsible for selecting the optimal hitting plan by screening TTB-hitting actions. Third, the control system or execution system is accountable for executing the instructions issued by the decision-making system (Oliveira et al., 2021; Payedimarri et al., 2021). Strength, speed, and accuracy are essential for TTB to be served or returned; thus, the trajectory prediction and timing must be precise.

Summing up, the research of TTR involves many fields, such as visual inspection (VI), intelligent decision-making, DL, and servo control. The present work mainly examines the software system of TTR to identify the motion state and trajectory of the TTB accurately.

### Related Research on Table Tennis Robots

The research of table tennis robots originated in the 1980s. The first table tennis robot developed can only serve and does not have the function of confrontation with human beings. Therefore, the table tennis robot could only serve as a companion for athletes to send different trackballs. In the future, with the progress of research and technology, a table tennis robot that can catch the ball will be developed gradually.

The robot table tennis game rules were first formulated by the University of Portsmouth in the United Kingdom in 1983. The rules stipulated that the table tennis table was 2 meters long and 0.5 m wide, slightly smaller than the usual table tennis table. The robot developed by Gerhard Schweitzer of a Robotics Institute in Zurich, Switzerland, won the championship of the competition in 1988 and the Hong-Kong Robot Ping Pong Competition in 1992.

In 1987, the Alcatel-Lucent Bell Labs of AT&T Inc. in the United States intensively studied the mechanical system, vision system, and control system of the table tennis robot. They adopted a PUMA 260 manipulator with six-Degree-of-Freedom (DOF) in a robot, which is more flexible when hitting the ball. It is a real table tennis robot because it can use the vision system to judge the position of table tennis. It has successfully realized a man-machine match for nearly 20 rounds.

The Toshiba Corporation of Japan has developed a seven-DOF manipulator that can hit a table tennis ball against the wall by itself. Picasso, a robot developed by the Rochester Institute of Technology in the United States, uses a five-DOF manipulator. However, the visual system cannot quickly capture the trajectory of the TTB to meet the requirements of the man-machine rally because the robot uses a series manipulator. The University of Adelaide in Australia designed a six-DOF series manipulator by imitating PUMA. Although it is more flexible than the previous TTR, it can move in a relatively small range.

In Japan, Fumio Miyazaki of Osaka University has developed a four-DOF table tennis robot based on binocular vision. Sensors are installed on the opponent's racket and elbow to detect the rotation direction of the ball, which enables the robot to receive the ball from different angles. A bipedal humanoid robot developed by TOSY Robotics JSC in Vietnam participated in the Tokyo International Robot Exhibition in 2007. It can play table tennis against a human being with flexible and accurate actions, which has become the focus of public attention.

Most of the existing manipulator systems adopt a series structure characterized by significant moments of inertia and relatively strong design load. The manipulator is often designed to adapt to the corresponding industrial production environment. Although it is convenient for research to use this industrial manipulator as the hitting execution system of table tennis robots, its shortcomings are also evident. The real-time playing of table tennis against humans requires a high response speed from the robot. The robot needs to recognize and predict the trajectory of the TTB in a short time and then order the manipulator to hit the ball quickly. Series manipulators cannot meet this requirement. Scholars employ the parallel robot as the actuator of the table tennis robot system because of its flexible dynamic performance. The parallel manipulator reduces the moment of inertia of the manipulator and improves its flexibility.

In the robot system, the operation of the inverse solution of the hitting point is cumbersome, bringing a significant challenge to the operation speed of the whole system. After obtaining the three-dimensional coordinate information of the table tennis ball, the vision system predicts the coordinates of the hitting point. The table tennis system needs to carry out the inverse solution according to this coordinate and calculate the rotation angle of each joint point. The manipulator has countless postures to strike a certain point. The inverse solution process aims to find the most reasonable hitting method with a particular scheme. The actuator of the seven-DOF manipulator needs to calculate the rotation angles of the seven joints, respectively, requiring a tremendous amount of calculation. Therefore, many scholars choose a relatively simple mobile guide rail to avoid this problem. This actuator has a larger hitting space than the industrial manipulator, simplifying the calculation and the cumbersome trajectory planning process.

## Research Model and Framework

### AI-Based TTB Trajectory Prediction

A table tennis ball has the characteristics of high speed, small volume, light weight, and fast rotation, resulting in high requirements for the real-time capability and accuracy of the vision system. Traditional target detection for TTB mainly adopts color segmentation, contour search, or multi-sensor methods. They can quickly respond to detection with simple calculations. However, the surrounding environment quickly affects the detection accuracy, such as illumination and background, leading to low detection accuracy. For example, the color segmentation algorithm sets the detection threshold according to the TTB color. Trajectory prediction tasks are mainly completed through physical modeling. The TTB rotation model features high-order non-linearity. Traditionally, the linear approximation algorithm is often used for modeling, with a relatively large deviation accumulating with the iteration, resulting in low prediction accuracy and less extendibility.

Under the current AI era, fusing AI, and robotics is the general trend to extend the development space and research value of robots while bringing more research possibilities to the TTR. In particular, the DL methods can exert their advantages against the existing problems in traditional ways. Yet, DL-based detection networks involve vast amounts of calculation and training data, thus complicating the issues. Therefore, aiming at the shortcomings of conventional TTB detection and trajectory prediction methods, this study integrates a DL method with strong generalization ability and anti-interference ability with the vision system to study the TTB rotation and predict its trajectory explicitly.

As a cutting-edge technology in the AI field, intelligent TTR is a scorching research topic worldwide (Nataraj et al., 2021; Sun, 2021). The ultimate task of an intelligent TTR is to play table tennis with people and even assist in professional training. According to the above analysis, the vision, decision-making, and control systems are subsystems of the intelligent TTR. Given that TTB is small and fly fast, the vision system shoulders the primary task of recognizing the TTB's dynamic state quickly and accurately. In some high-level competitions worldwide, many top table tennis players play at speeds up to over 20 m/s, and the rate of 5 m/s in general. The length of an ordinary table is 2.74 m, so it takes <0.5 s for a TTB to fly across the table (Pezzo and Beckstead, 2020). Thus, a TTR must detect and analyze the TTB dynamic state within 0.5 s, including TTB identification, trajectory prediction, and action planning. A TTR should have high-speed processing and calculation capability. Image recognition and trajectory prediction become the primary tasks of the vision system. In short, the main research goal and difficulty of the vision system of TTR are to capture and analyze TTB accurately and in time.

In addition to real-time trajectory prediction and target recognition, the vision system needs to provide a timely and reasonable response for the subsequent decision-making and control systems. According to international research, most visual systems can be divided into monocular visual systems and binocular visual systems based on the camera number (Geffen et al., 2020; Muto et al., 2021). According to the installation position, visual systems can be divided into ontological and external systems. As their names suggest, ontological systems install the camera inside the robot, which moves with the robot (Tomasevic et al., 2019). The outward vision system fixes the camera outside the robot so that the camera can only be calibrated without moving.

Most TTRs employ the binocular vision system because it can determine the target's spatial position. Compared with the binocular system, the monocular vision system has lower costs and lower calibration difficulty. Still, it needs to project a target to determine the target's spatial position (Tkatek et al., 2020; Jammeli et al., 2021). To some extent, it increases the difficulty of the algorithm and increases the requirements for the environment. Therefore, monocular vision systems are not as common as binocular vision systems.

Humanoid robots usually put the camera of their vision system inside. In contrast, other robots with robotic arms put the visual systems outside because it is difficult to use the ontology vision system; after all, the vision system will change with the robot's movement, and the camera needs to be recalibrated. Such a dynamic process makes algorithm implementation more difficult. Therefore, robots with good market influence usually choose an external vision system.

The primary function of robot servo planning is to identify the TTB and predict its trajectory by positioning the dynamic TTB according to the characteristics of a TTB using the camera.

**Figure 1:**
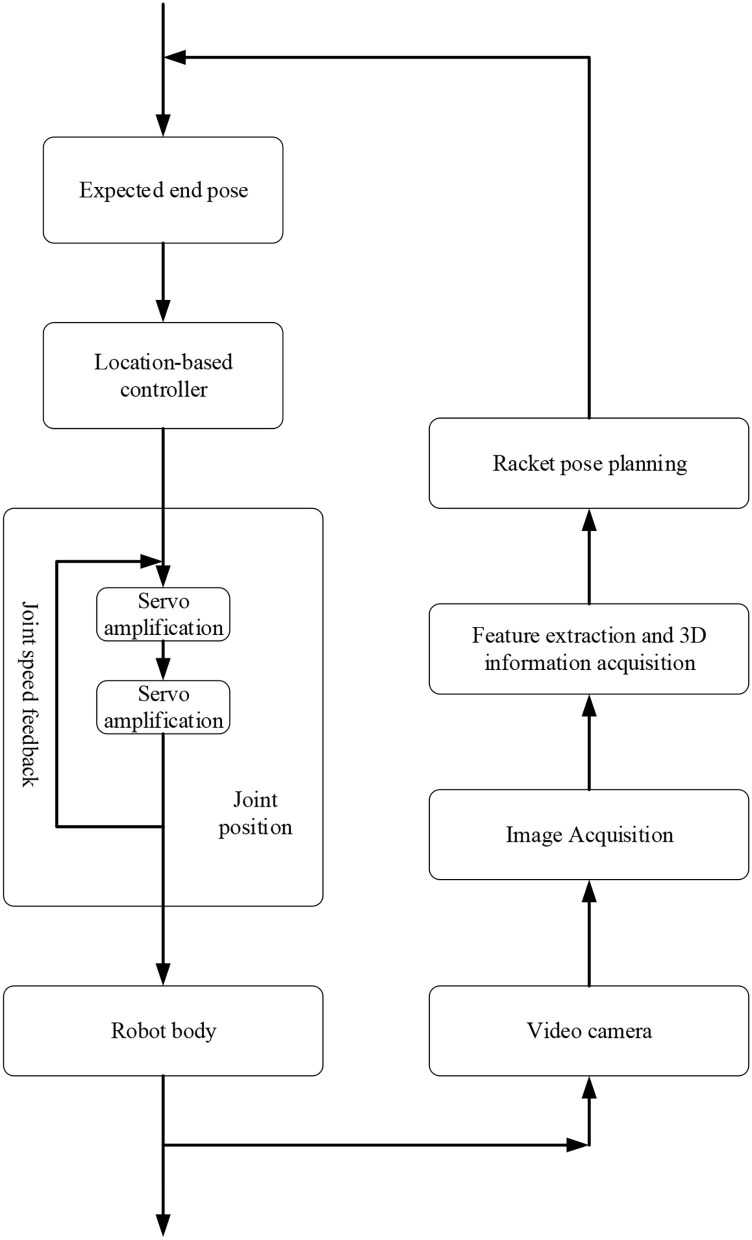
Visual servo control system based on position.

Here, the position-based visual servo control system is selected for TBR, as shown in Figure 1.

In Figure 1, the visual servo control system adjusts the internal and external parameters of the camera. The target's spatial pose is calculated according to the camera image. Another camera can photograph the TTB's edge, calculate the edge pose of the TTB through feature extraction and other operations, and compare it with the expected posture. The pose deviation of the TTB edge is calculated through two differentiations. Then, the pose deviation of each side of the TTB is transformed into the base coordinate system.

### Correlation Between TTB Rotation and Trajectory Based on Neural Network

In a competition, TTB is often rotating. Thus, the critical point of trajectory prediction of the TTB lies in choosing prediction methods. Here, the rotating state of TTB is divided into two categories for detection. The first category is identifying the TTB by marking TTB. Once labeled, the system can directly calculate the rotation of TTB by calculating the movement relative to the center of TTB (Salvatore et al., 2021). In the second category, the TTB trajectory will be collected and analyzed, and then the dynamic state of TTB is reversed according to the trajectory. However, the research in this field is only based on the TTB rotation to judge its trajectory. Such technology is impossible to accurately judge the exact amplitude, direction, pose, and rotation speed (Lin et al., 2021).

The trajectory of the table tennis ball in the air is expressed by three kinematic parameters: spatial coordinates, velocity, and acceleration. In the whole process of ball movement, these parameters will change from time to time according to the different movement times of the ball (Glassman et al., 2021). Then, a trained deep neural network (DNN) is selected here. Nine TTB parameters are input into DNN to output the TTB landing point. In actual competitions, the player's prediction based on TTB trajectory is also the primary determinant of the ball's landing point. Accordingly, the DNN aims to calculate the landing point data.

The TTB trajectory analysis is mainly carried out through SIMI Motion in this experiment. This software system can analyze various sports and movements based on three-dimensional (3D) video, typical in sports technology analysis and teaching. SIMI Motion uses multiple cameras to synchronize the target motion, the multi-dimensional 3D frame for calibration, and manually marks the joint points. Meanwhile, SIMI Motion can obtain the moving object's two-dimensional (2D) and 3D data to calculate the coordinate, speed, acceleration, and angle between the marked points. This experiment uses SIMI Motion to synchronize two cameras to obtain the TTB trajectory data. First, the calibration is completed by photographing the multi-dimensional 3D frame of two cameras and manually marking the white dots on the 3D structure. Then, the synchronization light is used to synchronize the time of the two videos. Afterward, the SIMI Motion is used to manually mark and determine the spatial position of the TTB in each frame, thereby obtaining the 3D data of the TTB trajectory to calculate the TTB rotation speed, a vector with size and direction. Precisely, the high-speed camera captures the initial rotation track of the TTB transmitted by the TTR's serving module and calculates the number of image frames required for the LOGO to rotate one circle to determine the rotation speed of the TTB. The TTB's initial state (when TTR fires TTB) is recorded as the frame *n*_1_ in the video, and the TTB state after one-circle rotation is denoted as the frame *n*_2_. The frame rate of the high-speed camera is 3,000 frames/s; thus, the TTB rotation speed is $\frac{3000}{n_{2}-n_{1}}$ R/S. Subsequently, the rotation direction data is obtained through the TTR's service module. The TTR's service module serves the rotating ball through two pulleys and controls the size and direction of rotation by controlling the speed and direction of the upper and lower pulleys. This experiment tests the rotation type of service from the service modules. There are nine rotation types of service: topspin, backspin, left-sidespin, right-sidespin, left-side topspin, right-side topspin, left-side backspin, right-side backspin, and without spin, as detailed in Table 1.

**Table 1:** Description of the table tennis robot (TTR)'s serve.

**Type of service**	**Speed of upper and lower pulleys**	**Horizontal angle of the pulley**
Topspin	The upper pulley is fast and the lower pulley is slow	90°
Backspin	The upper pulley is slow and the lower pulley is fast	90°
Without spin	The speed of the upper and lower pulleys are the same	90°
Left-Sidespin	The upper pulley is fast and the lower pulley is slow	0°
Right-Sidespin	The upper pulley is slow and the lower pulley is fast	0°
Left-Side topspin	The upper pulley is fast and the lower pulley is slow	−45°
Left-Side backspin	The upper pulley is slow and the lower pulley is fast	45°
Right-Side topspin	The upper pulley is fast and the lower pulley is slow.	45°
Right-Side backspin	The upper pulley is slow and the lower pulley is fast.	−45°

As listed in Table 1, the TTR can only implement nine types of service due to the limitations of the service module. The horizontal angle of the pulley determines the TTB rotation direction. Because the outlet of the service module is horizontal, the fired TTB's initial velocity is in the horizontal direction. Then, the TTB speed can be obtained through SIMI Motion analysis. Additionally, the initial position of the ball outlet is known. Then, nine accurate initial values can be obtained, including the initial position *x* with direction coordinate (*x*); initial position *y* with direction coordinate (*y*); initial position *z* with direction coordinate (*z*); initial position *x* with direction velocity (*v*_*x*_); initial position *y* with direction velocity (*v*_*y*_), initial position *z* with direction velocity (*v*_*z*_); initial position *x* with direction rotation size (ω_*x*_); initial position *y* with direction rotation size ω_*y*_; initial position *z* with direction rotation size ω_*z*_. The origin of the coordinate system is set at the table tennis table's midpoint, where the service module is located. The abscissa extends along with the table horizontally, the vertical coordinate extends longitudinally, and the vertical coordinate is perpendicular to the table. A total of 171 effective balls are served by the service module, covering nine types of service, and nine initial and landing point values of all balls are obtained. The falling point data are the coordinates on the table, so the ordinate is 0. Through the experimental data collection, to get the accurate initial position coordinates, the accurate initial velocity (including speed and direction), and the accurate rotation velocity, the nine initial data are input into the DNN to output the precise landing point coordinates. Finally, the DNN algorithm explores the correlation between the input and output information.

In this experiment, Matlab pattern recognition is adapted for the trajectory prediction of TTB with different velocities and rotations.

The neuron nodes in the input layer have nine dimensions marked as “a.” Neural nodes in the output layer have two numerical dimensions marked as “b.” These are the abscissa and ordinate of TTB. The number of nodes in the hidden layer is determined after confirming the input and output neurons according to Equation (1).

(1) $$\begin{array}{r} F=\sqrt{0.43ab+0.12b^{2}+2.54a+0.77b+0.35}+0.51 \end{array}$$

The values of *ab* are substituted into Equation (1) to solve the number of hidden nodes as 6.

**Figure 2:**
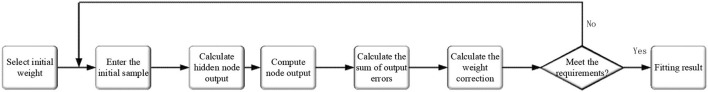
Fitting process of neural networks.

Then, the Non-linear Neural Network algorithms, such as Levenberg-Marquard (LM), Budgeted Rooted (BR), Backpropagation Neural Network, and Scaled Convergence Gradient (SCG) are used to fit the data using the Matlbe neural network toolkit, as displayed in Figure 2.

### TTB Test Experiment Based on DL

The experiment begins after the preliminary preparations (Hildebrand et al., 2021; Zhang et al., 2021). The DNN used in the experiment is designed by Matlab toolkit, specified as follows.

Step 1. Collect data and import the input and output data into the database.
Step 2. Conduct simple data processing, including normalization, adjustment, and reconstruction. The regularization equation is:

(2) $$\begin{array}{r} M=\frac{m-m_{\min}}{m_{\max}-m_{\min}} \end{array}$$

The input data is disturbed before input to improve the generalization ability of neural networks and avoid the relative concentration of data in the same service spin mode.

Step 3. Construct the initial structures of input, output, hidden layer nodes, and the transfer function of the neural network.
Step 4. Set training time, target, error, and other parameters. Start training. Add weight correction parameters.
Step 5. Input the data to be tested after training.

**Figure 3:**
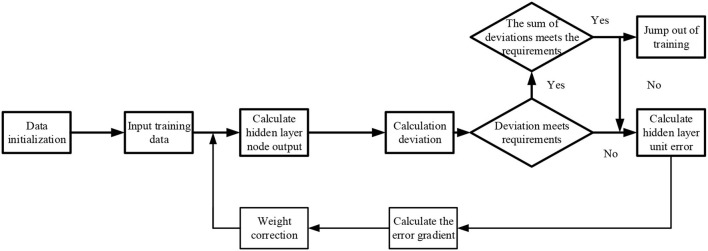
Table tennis ball (TTB) test based on DL algorithm.

Figure 3 illustrates the experimental process.

### TTB Detection Based on Feature Fusion Network

#### FFN

The deeper a neural work is, the more times it scales down the original input image; thus, when locating small targets, some DNNs might get awkward performance, such as very low detection accuracy. In other words, every time the convolution kernel extracts image features, the feature map will shrink down by some ratio while the rich semantic information continues to be strengthened. Such operation is conducive to object detection and classification tasks, but the object's location information will be gradually discarded. Therefore, fusing the underlying feature information in the convolution downsampling process is necessary to enhance the network's ability to detect and locate small targets.

**Figure 4:**
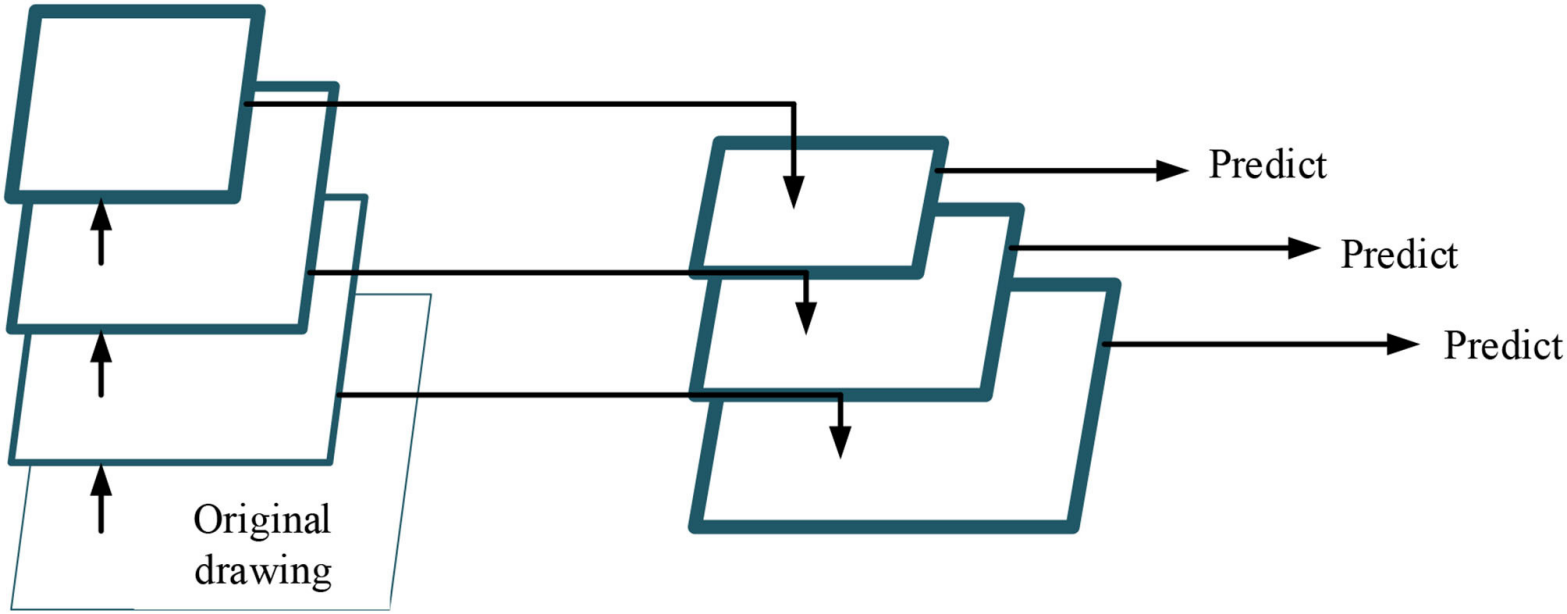
Feature pyramid networks (FPN) structure.

Earlier, neural networks need to input fixed-size images. Such operations as folding and flipping are often used in detecting photos with changed sizes, leading to information loss, which limits the accuracy of network recognition. In particular, feature pyramid networks (FPNs) can combine humble features lost in the original downsampling process. FPN will not increase model calculation substantially. On the contrary, it downsamples the semantically-rich upper feature layer in the top-down hierarchical network structure and then stacks and fuses the sampling results with the feature map of the same size, significantly improving the model's performance in small objects detection. Each layers' output can detect the type and position of objects. Figure 4 displays the structure of FPN.

**Figure 5:**
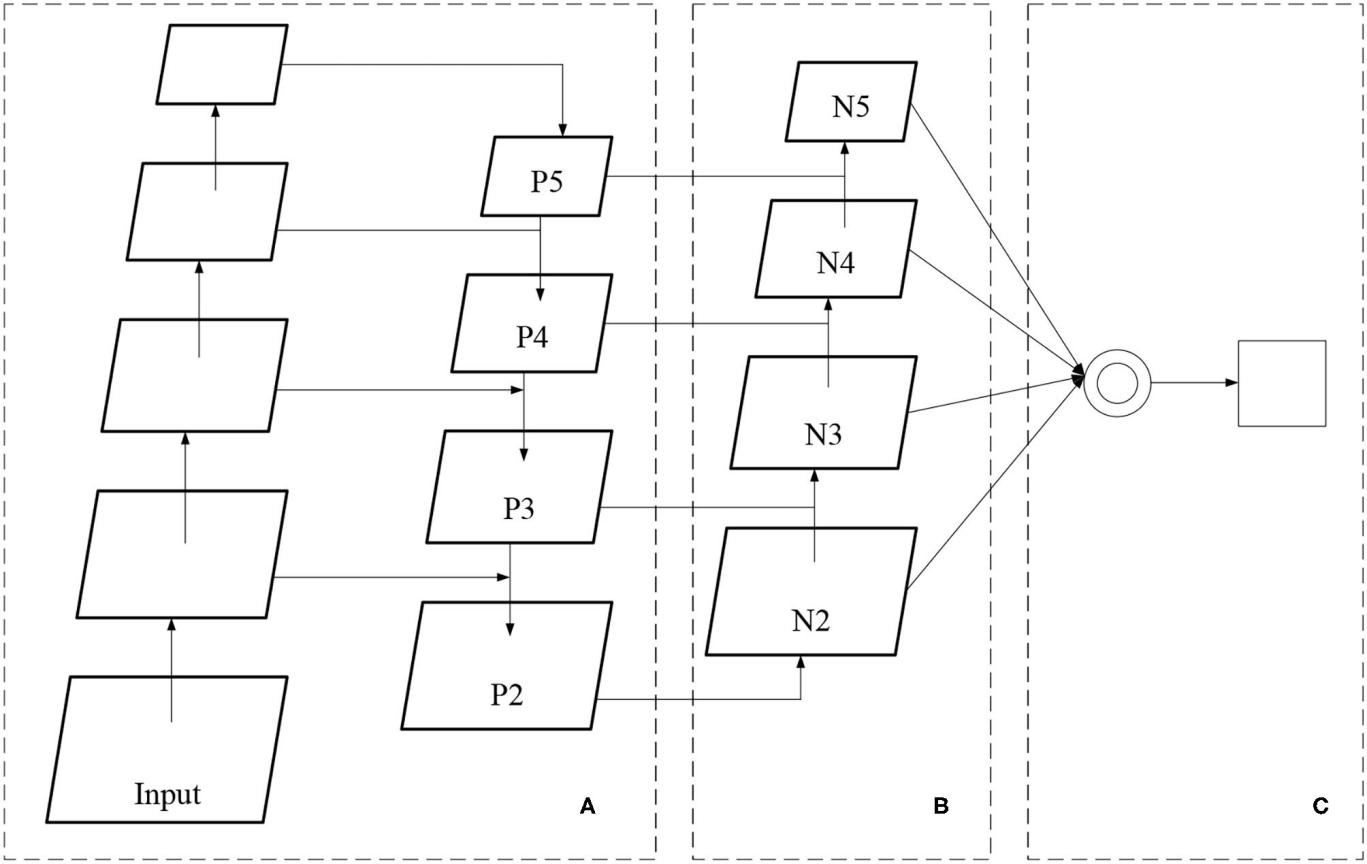
Feature fusion network (FFN)'s structure.

Two horizontally linked processes go through the FPN structure: bottom-up and top-down approaches. The bottom-up process is the forward propagation process when the feature map gradually shrinks with convolution kernel calculation. In contrast, the top-down process upsamples the feature map, and the horizontal link uses a 1^*^1 convolution kernel to fuse the feature map of the same size generated by bottom-up and top-down processes. In this way, the position details at the network bottom can train the network with accurate location information while simultaneously learning the target features, especially for small object detections. This paper improves FPN by adding a bottom-up connection, as shown in Figure 5.

A convolutional neural network (CNN) usually uses a single random feature layer (sometimes the last) to detect and locate targets. Each feature layer has its unique function for the target detection task. In Figure 5, the feature layer is followed by the adaptive pooling layer in area C. Then, the feature layers in B are merged into a feature layer of the same size. Then, the final feature layer of target detection is obtained by max-pooling feature fusion. Such a design can combine the feature information of each feature map to significantly improve the position detection ability of the network to small targets.

#### Target Detection Network

**Figure 6:**
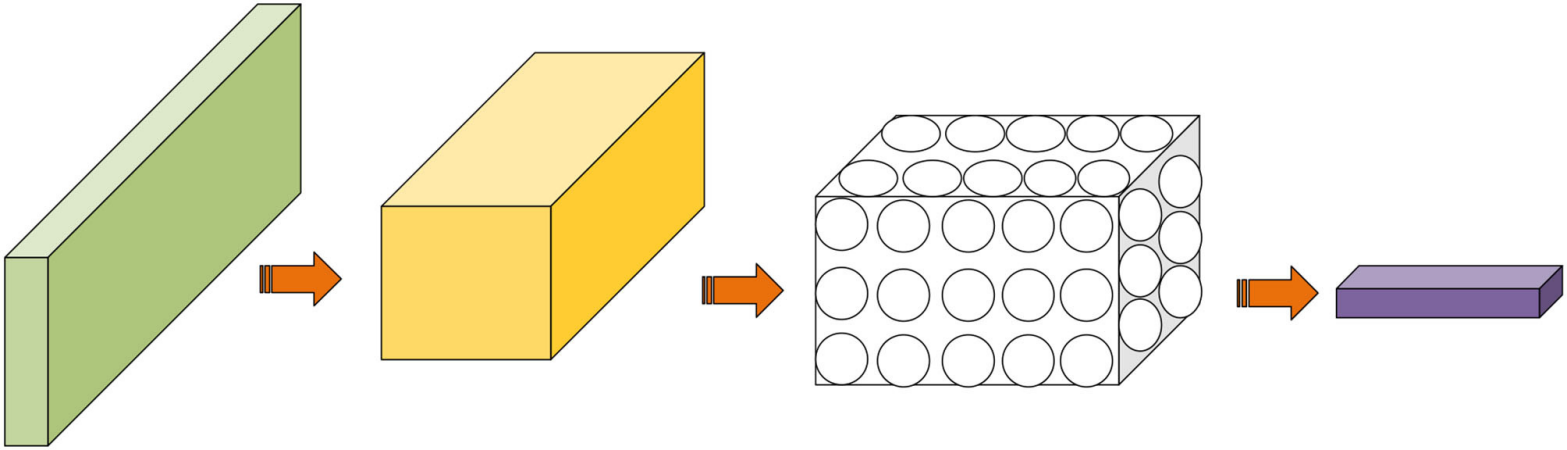
Convolutional neural network (CNN)'s structure.

Convolutional neural network is a supervised learning DNN, including convolution and pooling layers to extract input image features, an activation function to increase the non-linear ability of the network, and a fully connected layer to realize target detection and classification. At the same time, CNN can share weights, simplify network parameters, and avoid overfitting. Figure 6 shows the structure of CNN.

The convolution layer is the most critical structure of CNN. It comprises convolution kernels of various sizes and depths. The network depth mainly refers to the number of convolution layers. During network initialization, the size and depth of the convolution layer will be set. Afterward, during network training, the network parameters repeatedly learn the data features, and backpropagation is used to optimize these parameters. The image RGB value is constantly multiplied by the network weights to extract the image features during network transmission. The convolution layer can extract features and reduce the image dimensions. CNN employs valid padding (set to zero) to output images with the same dimensions as the original image. Different sliding steps can be set to reduce the image dimension. The smaller the sliding step size is, the fewer characteristic images are obtained. Generally, the richer the features to be extracted, the smaller the sliding step. Equation (3) indicates the output dimension of the convolution layer.

(3) $$\begin{array}{r} \begin{array}{c} W_{2}=\left(W_{1}-F+2P\right)/S+1\\ H_{2}=\left(H_{1}-F+2P\right)\\ D_{2}=K \end{array} \end{array}$$

In Equation (3), *W*_1_ and *H*_1_ represent the height and width of the input image. *F* is the size of the convolution kernel. *P* denotes the value of padding. *S* indicates the step size when the convolution kernel performs convolution operation. *H*_2_ and are the length and width of the output dimension.

Following the convolution layer is generally the pooling layer whose scanning process is the same as that of the convolution layer. The pooling layer can reduce the resolution of the feature map, thereby simplifying the network parameters. Generally speaking, the pooling layer involves two operations: max pooling based on operation and mean pooling based on average. Max pooling is most commonly used and realized by taking a receiving domain's maximum value. Mean pooling adds all selected pixel values and then averages them.

A normalization operation often follows the pooling layer to avoid the impact of nodes with large data values on classification. The purpose of normalization is to contain the input within an acceptable range [0, 1]. In CNN, the data normalization can be conducted before or after the fully connected layer or elsewhere, depending on the network structure.

Finally, the classification task is realized by the fully connected layer, which is mainly composed of convolution kernels of different sizes. The softmax layer can classify the fully connected layer's output. Additionally, a fully connected layer needs to optimize a substantial number of parameters, accounting for almost 80% of the network parameters. Some scholars have used the global average pooling to replace the fully connected layer, enhancing the network detection ability.

The first research on CNN can be traced back to the twentieth century. Common CNN structures include convolution, pooling, activation, and fully connected layers. Previous feature extraction networks mainly reduce the data dimension through a series of convolution downsampling and extract or sort out valuable features for subsequent use. In downsampling, the model captures and learns the object features. These semantically rich feature layers can enable the model to judge the image types. Still, the increase of the network depth also trades off the position information of the object in the original image, thus bringing great difficulties to the detection of small entities.

In the forward propagation of the network, Mean Square Error (MSE) is generally used to measure network loss. Suppose *c* classes and *N* training samples for a classification problem. Then, Equation (4) holds.

(4) $$\begin{array}{r} E^{N}=\frac{1}{2}\overset{N}{\underset{n=1}{\sum }}\overset{c}{\underset{k=1}{\sum }}{\left(t_{k}^{n}-y_{k}^{n}\right)}^{2} \end{array}$$

In Equation (4), $t_{k}^{n}$ represents the dimension *k* of the sample *n*. $y_{k}^{n}$ means the *k*-th output of the network. The production of the multi-classification task often differs according to the different activation functions. Generally, only the output node corresponding to the input is positive, and the bits or nodes of other classes are 0 or negative. In the backpropagation of a sample, the error of the sample *n* is calculated according to Equation (5).

(5) $$\begin{array}{r} E^{n}=\frac{1}{2}\overset{c}{\underset{k=1}{\sum }}{\left(t_{k}^{n}-y_{k}^{n}\right)}^{2}=\frac{1}{2}||t^{n}-y^{n}|{|}_{2}^{2} \end{array}$$

In the traditional fully connected neural network, it is necessary to calculate the partial derivative of the loss function about each weight of the network according to the rule of backpropagation. Here, *I* represents the current layer. Equation (6) describes the output of the current layer:

(6) $$\begin{array}{r} x^{l}=f\left(u^{l}\right),u^{l}=W^{l}x^{l-1}+b^{l} \end{array}$$

There are various output activation functions, such as Sigmoid or Tanh. Sigmoid compresses the output to [0, 1], so the final output average ~0. Therefore, if the mean of training data is normalized to 0 with variance 1, the convergence can be accelerated.

The backpropagation of CNN can be called the sensitivity basis of each neuron, meaning that the error changes as much as the basis changes. Equation (7) expresses the sensitivity basis.


(7) $$\begin{array}{r} \frac{\partial E}{\partial b}=\frac{\partial E}{\partial u}\frac{\partial u}{\partial b}=\delta \end{array}$$

When $\frac{\partial u}{\partial b}=1$, there is Equation (7); in other words, the sensitivity and error of the basis are equal to the derivative ($\frac{\partial E}{\partial u}$) of all inputs of a node. This reciprocal transformation allows errors in the upper layer to propagate back to the lower layer. The backpropagation process can be described as Equation (8).

(8) $$\begin{array}{r} \delta^{l}={\left(W^{l+1}\right)}^{T}\delta^{l+1}Of^{\prime }\left(u^{l}\right) \end{array}$$

In Equation (8), *O* is to multiply each corresponding element in the matrix. Equation (9) expresses the sensitivity of neurons.

(9) $$\begin{array}{r} \delta^{L}=f^{\prime }\left(u^{L}\right)O\left(y^{n}-t^{n}\right) \end{array}$$

Delta rule is generally used in weight updating. The input delta is used for amplification and contraction for each neuron input. Then, the input and the sensitivity of the *l*-th layer are cross-multiplied to represent the derivative of the error to the weight matrix of the *l*-th layer network. The final weight update needs to be multiplied by a negative learning rate, as shown in Equation (10):

(10) $$\begin{array}{r} \begin{array}{c} \frac{\partial E}{\partial W^{l}}=x^{l-1}{\left(\delta^{l}\right)}^{T}\\ \Delta W^{l}=-\eta \frac{\partial E}{\partial W^{l}} \end{array} \end{array}$$

In Equation (10), η is the specific learning rate corresponding to each weight. The operation of the convolution layer is generally referred to as downsampling. During downsampling, the image size is continuously reduced to make the abstract features of classification tasks more evident. Compared with downsampling, upsampling can enlarge the image resolution, and the resolution-enlarged image can exceed the original image in quality.

Interpolation is usually used for upsampling the input image, that is, inserting some new elements into the original image. The implementation methods are mainly divided into three categories.

(1) The nearest neighbor interpolation, also known as zero-order interpolation, uses the gray value of the image for interpolation. For a pixel in the original image, the gray value of the nearest input pixel value is inserted into the transformed image. The advantage of this method is that the calculation is simple and easy to implement, but the accuracy is low. It will cause contour or texture blurring.

(2) Deconvolution transforms the input low-dimensional features into high-dimensional features. However, deconvolution mainly restores the size of the image without completely restoring the original quality. For example, under a step size of 1, to convert a 2^*^2 feature image into its original 4^*^4 quality, zero padding will be performed on elements with padding = 2. Then a convolution kernel of 3^*^3 will be used for convolution operation. Finally, a 4^*^4 image is generated. Zero padding will be performed around each element when the step size is >1; then, the same operation with a step size of 1 will be served.

(3) Reverse pooling is mainly divided into max pooling and average pooling. The former needs to record the maximum value position in the feature map, and the other positions are filled with 0. The average pooling generally uses adjacent elements for padding.

The CNN-based target detection algorithms are mainly divided into candidate box-based two-stage and regression-based one-stage networks. In the two-stage network, candidate frame extraction and target detection are divided into two parts. Specifically, the target's candidate box needs to be extracted, based upon which the target detection is carried out. One-stage network cancels the target candidate box extraction of the two-stage network and features a single-step prediction. The one-stage network outputs a five-dimensional prediction result, including the object category, the center point position of the object, and the size of the prediction box, with a faster detection speed than a two-stage network.

**Figure 7:**
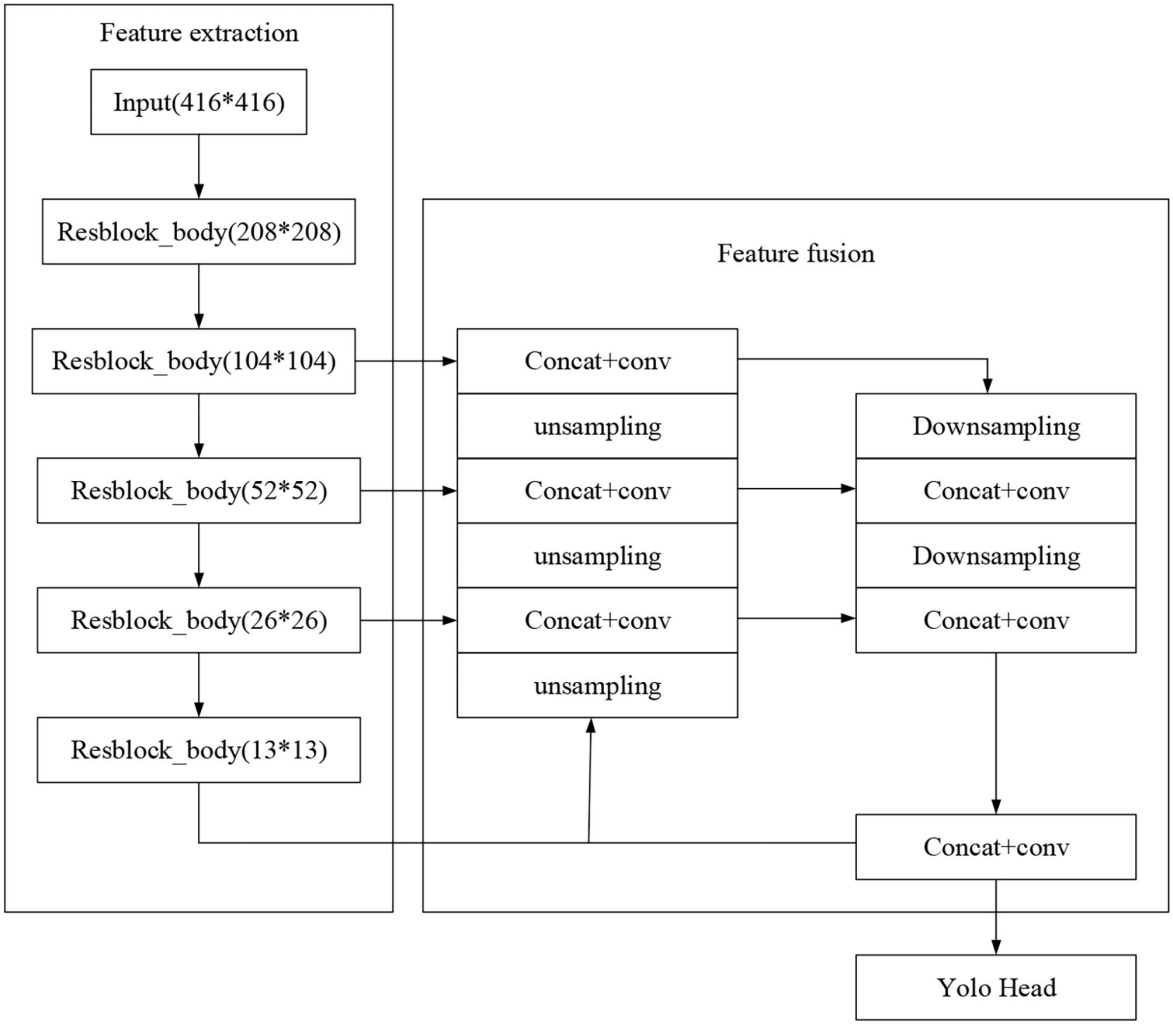
Network structure diagram.

Then, this study combines CNN with cross-layer link structures in the feature extraction network to solve the existing problems in the above methods. It constructs an FFN, as demonstrated in Figure 7.

**Figure 8:**
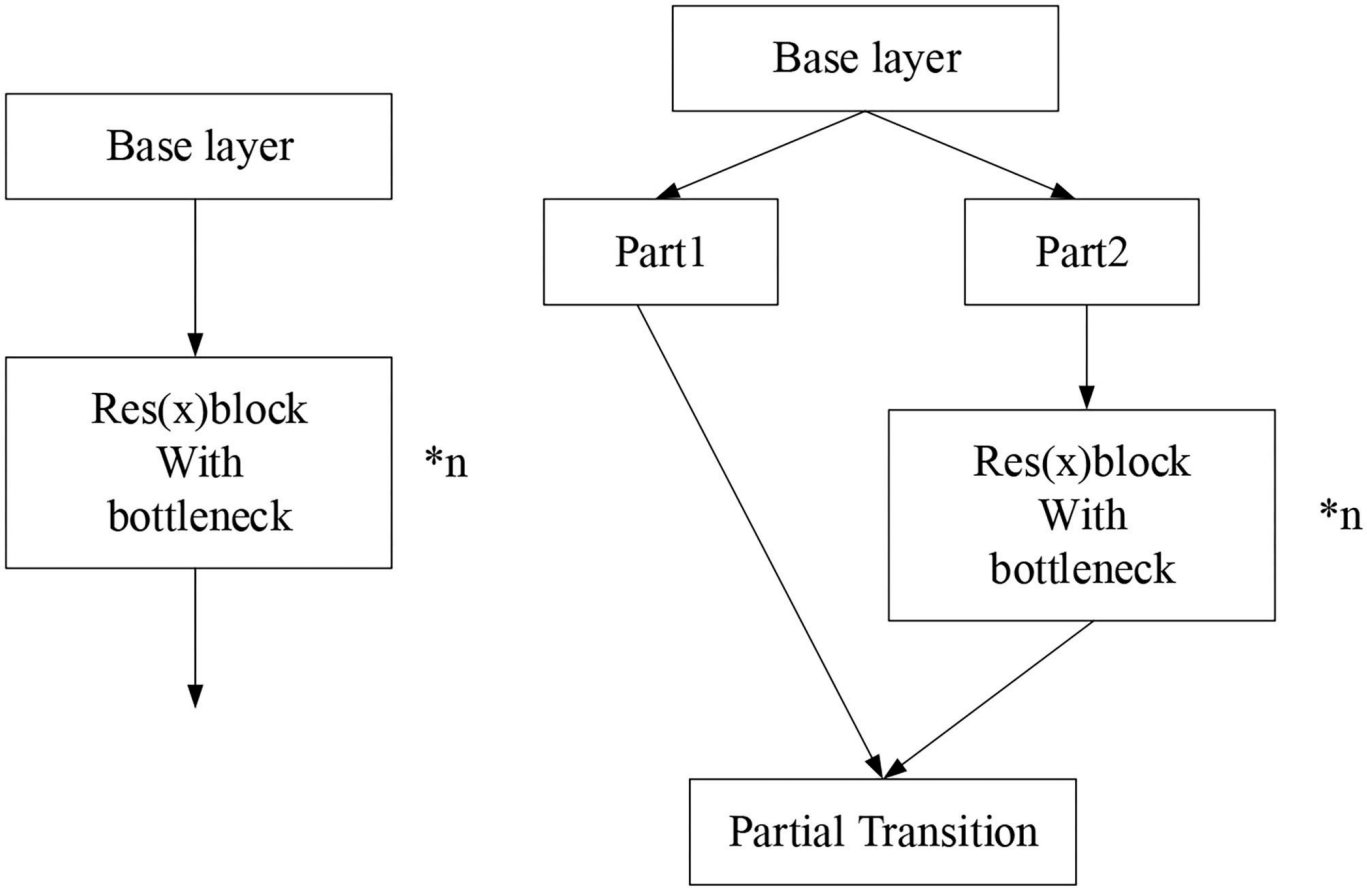
Cross-layer link structure.

The complex and extensive parameters result in the slow detection speed of neural networks. Therefore, reasonably lightening the network structure and streamlining network parameters is the key to improving the network detection speed. Accordingly, the present work combines the CNN and cross-layer link network in the feature extraction design. The input feature map is divided into two parts. One part is extracted through the residual network. The other part is directly concatenated and stacked with the output of the residual network after feature integration through a 1^*^1 convolution kernel. In this way, a large concatenation residual network is constructed. Due to information loss during feature extraction by convolution kernel with a deepening network structure, such concatenation residual network can recover the lost information during network learning and strengthen feature extraction. The concatenated feature map will pass through a transition layer to prevent the network from learning repeated gradient information during backpropagation, optimizes the network gradient propagation, and reduces the convergence time of the network. Figure 8 details the cross-layer link network.

The convolution kernel is set as follows. Res *block*_*body* is composed of 1^*^1 and 3^*^3 convolution kernels. The 1^*^1 convolution kernel reduces the network parameters by compressing (reducing) the dimensionality of the feature map, and the convolution kernel of 3^*^3 is used for feature extraction. Such a design can effectively streamline network parameters and increase its nonlinear ability. In forward propagation, each Res *block* stacks the input of this layer with the twice-convolutioned output. If the input is *x*, *x* + Res *block*(*x*) is the output. Such a structure constructs a basic residual block. Res *block*_*body* is the core part of building the whole network. Ordinary residual networks simply stack Res *block*. By comparison, the cross-layer link networks introduce an enormous residual edge Part 1 in the stacking process of fast residual. Part 2 continuously extracts the features of the input image, while Part 1 directly connects the input to the output of Part 2 with a small amount of processing.

Common activation functions include sigmoid, ReLU, tanh, leakyReLU, and Mish; Sigmoid can easily lead to gradient dispersion. When the ReLU is negative, the network neurons stop learning, and Mish will increase computation significantly. By contrast, leakyReLU is much more robust against these shortcomings. Therefore, this section adopts the leakyReLU as the activation function to simple calculation, preventing gradient explosion and dispersion. Equation (11) indicates the leakyReLU:

(11) $$\begin{array}{r} f\left(x\right)=\max\left(ax,x\right) \end{array}$$

### Design of the Physical Robot System

**Figure 9:**
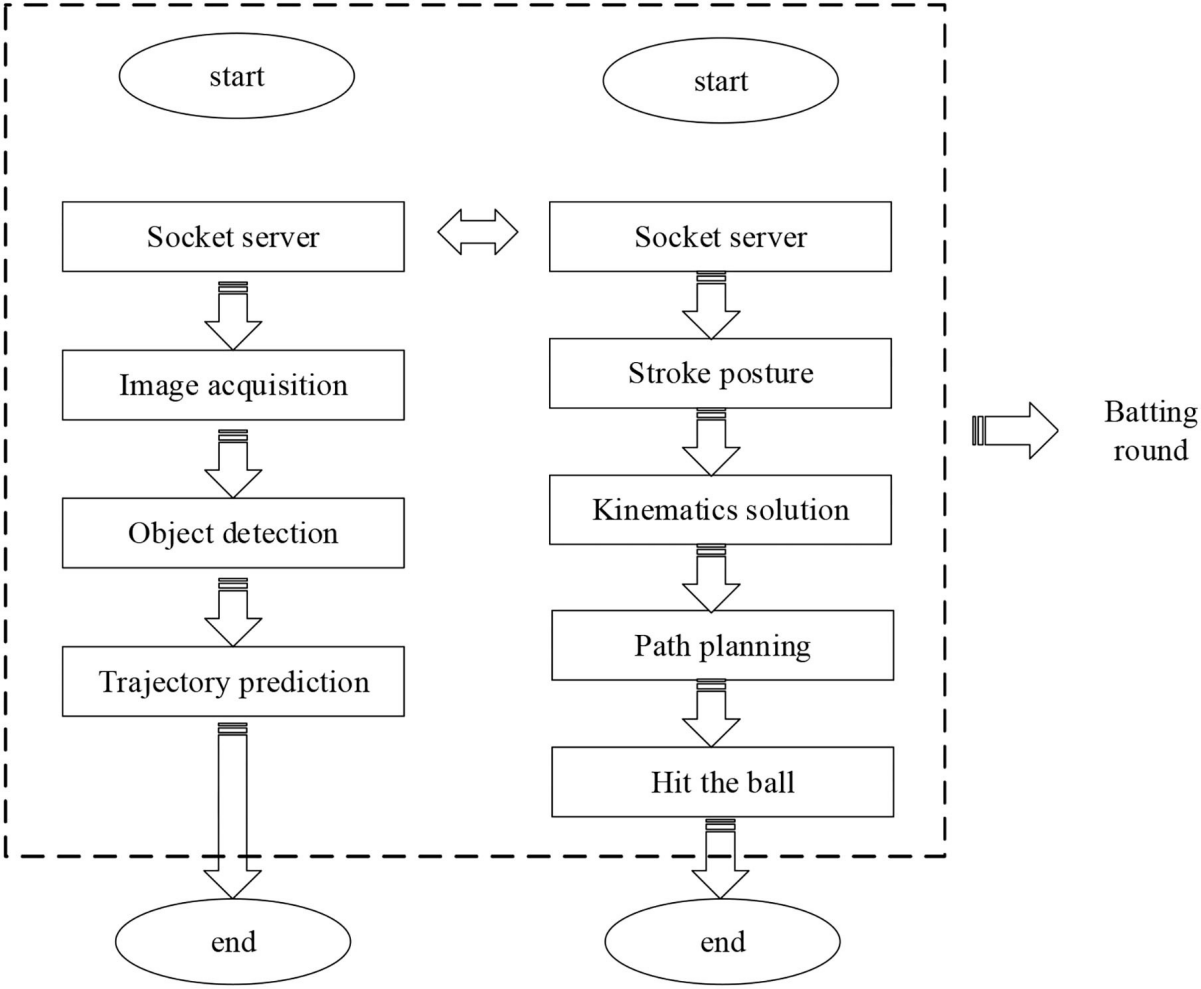
Hitting flow of the table tennis robot (TTR) system.

The seven-DOF KUKA manipulator system adopts a communication module of a 32-bit system. The vision system used here is designed in the 64-bit operating system. Therefore, the programs of the two parts should be designed into two modules separately. On the contrary, the two modules need to be connected to realize signal transmission in practice. The abstract layer encapsulates the Transmission Control Protocol/Internet Protocol (TCP/IP) family and only provides several interfaces to the application layer. It only needs to call these interfaces to realize real-time information communication when in use. Figure 9 displays the TTR system designed here.

**Figure 10:**
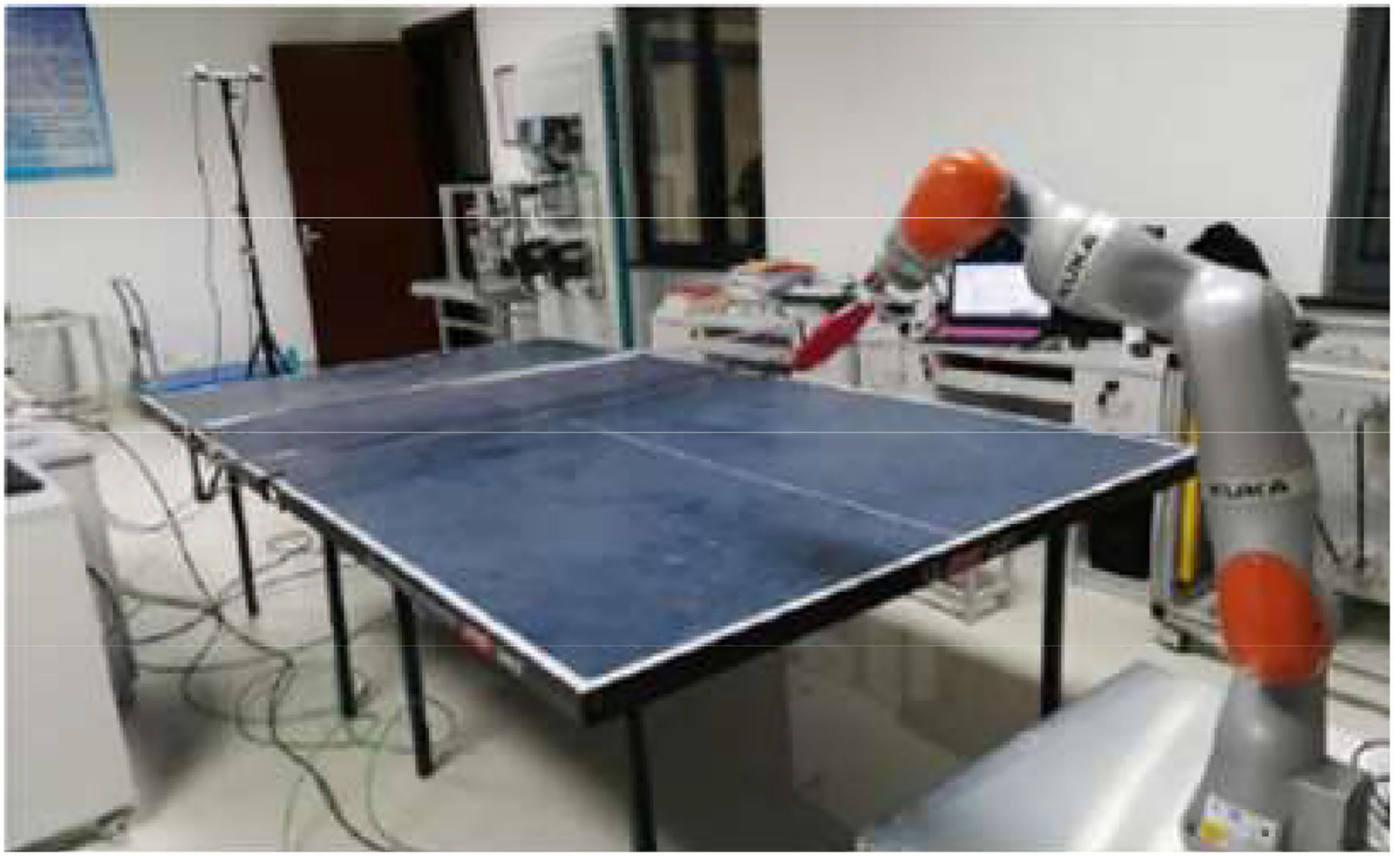
Physical system of the 7-DOF table tennis robot.

Figure 10 illustrates the physical design of the 7-DOF robot established here.

## Results and Discussion

### Results of the Performance of DL-Based TTR

**Figure 11:**
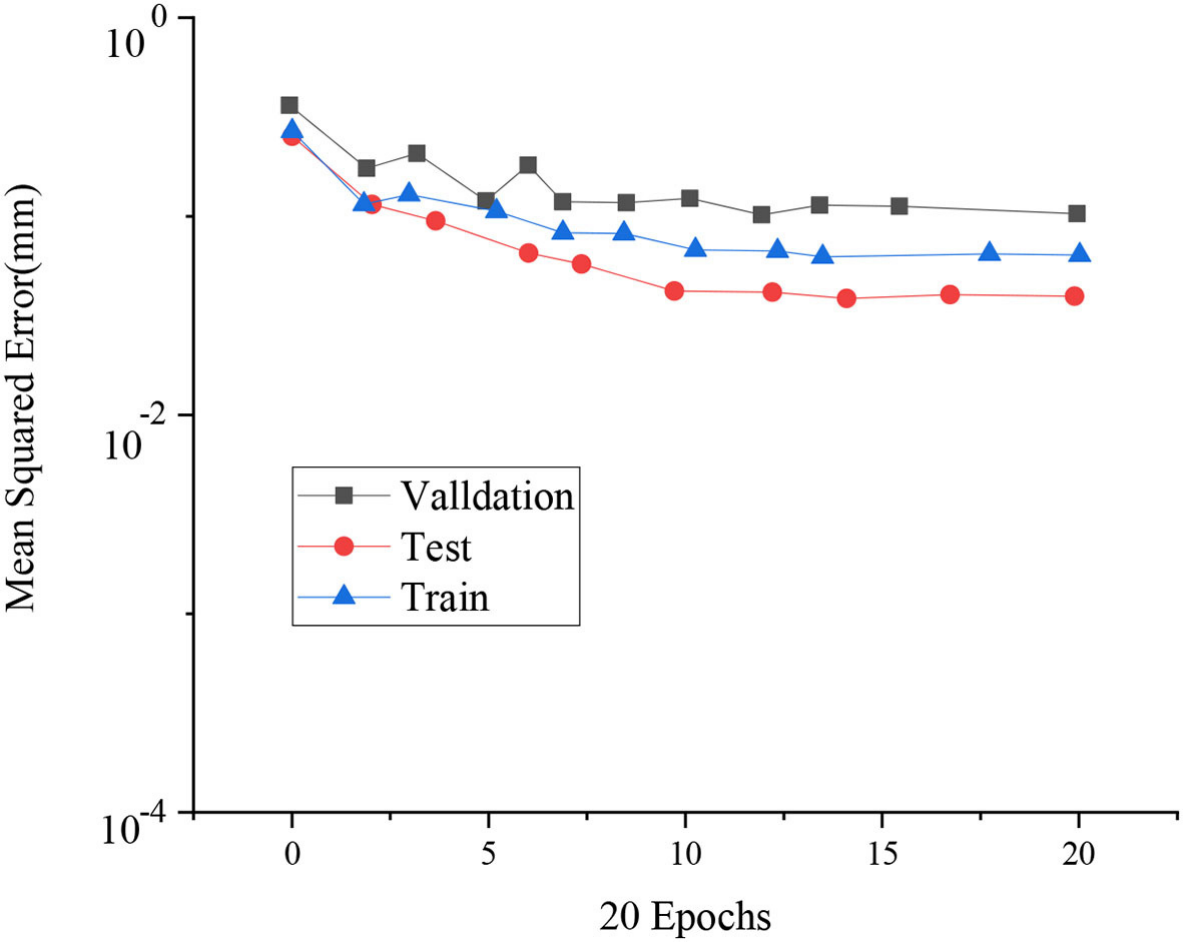
MSE of fitting training based on LM algorithm.

Figure 11 reveals the results of MSE of fitting training based on the LM algorithm.

The three curves in Figure 11 represent the verification performance after different data sets are input into the model. The training time is 20, and the MSE of the test data set reaches the minimum (0.057) when trained 14 times. Overall, the error of the test set is smaller than the training set and the verification set.

**Figure 12:**
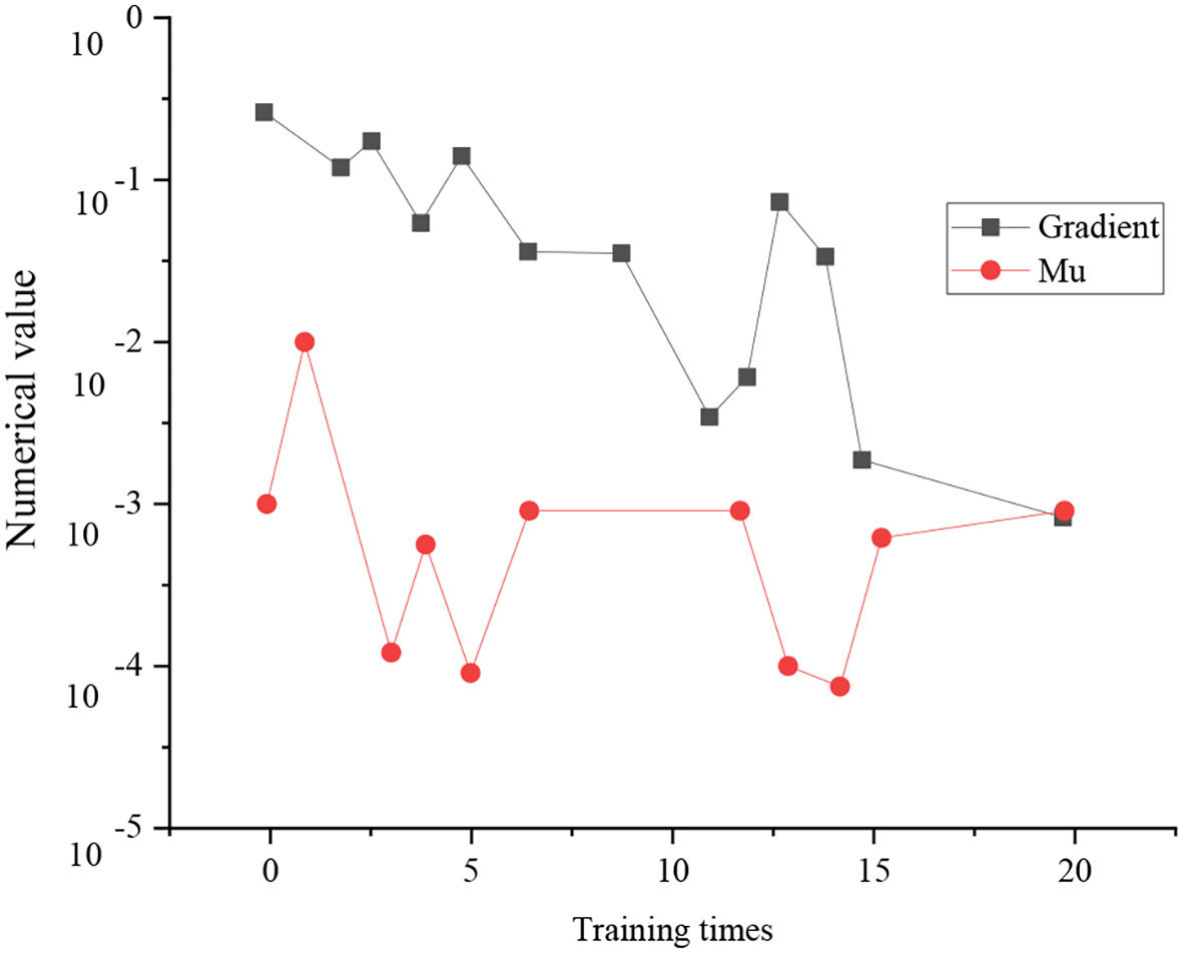
Training state of neural network based on LM algorithm.

Figure 12 displays the training state of the neural network based on the LM algorithm.

As shown in Figure 12, there are two indexes to judge the training state of neural networks, namely, the training gradient of the LM algorithm and Mu value. Obviously, with the increase of training times, the overall training gradient of neural networks tends to decline. After 20 times of training, the gradient comes to 0.001598. The second index is the Mu value, a parameter in the training model algorithm. According to the Mu value, the overall trend first increases with the training times and then declines. After 20 times of training, the Mu value is 0.001.

**Figure 13:**
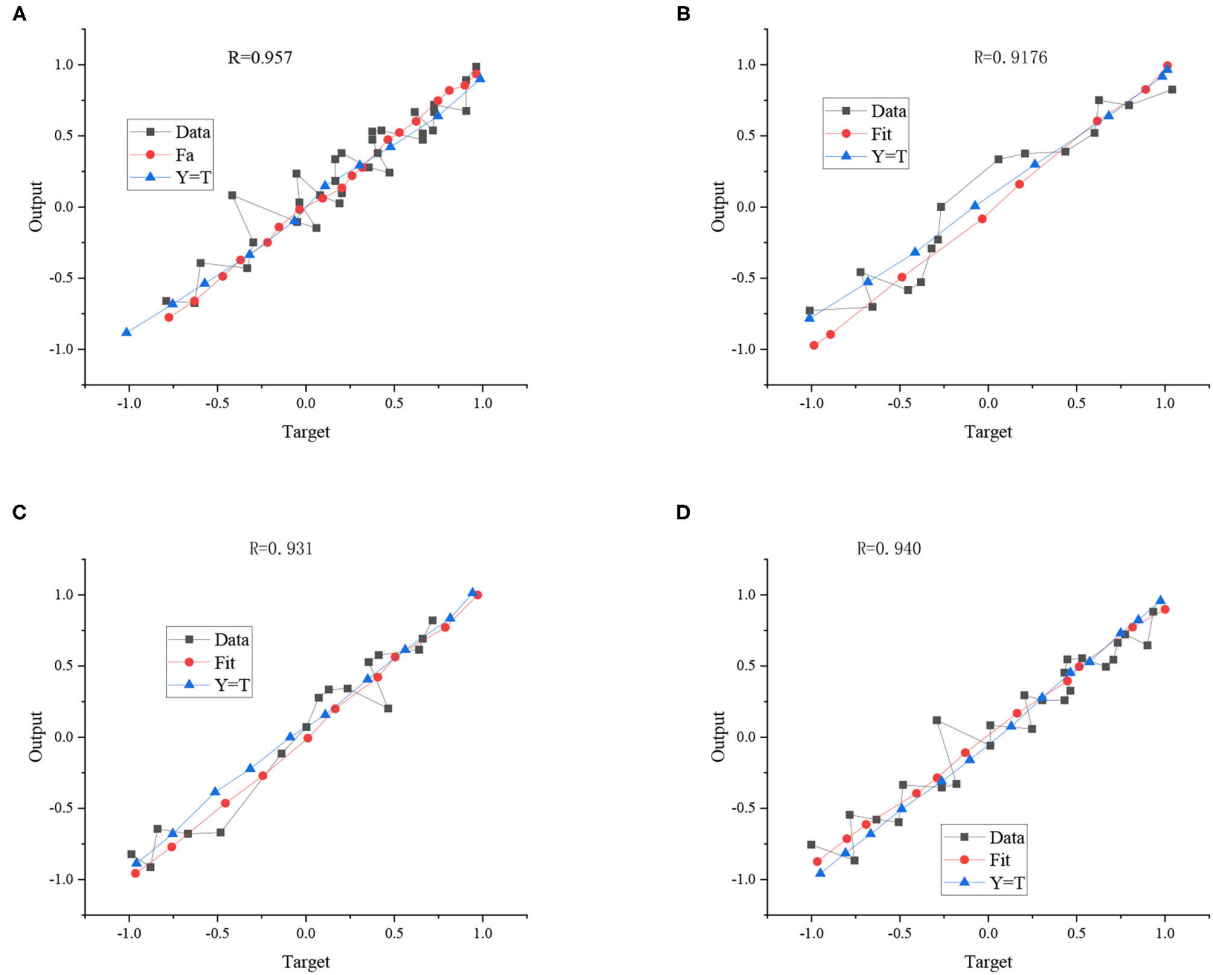
LM algorithm fitting results: **(A)** training set, **(B)** verification set, **(C)** test set, and **(D)** total input set.

Figure 13 shows the fitting results of the LM algorithm.

In Figure 13, R represents the correlation coefficient (CC) between the expected and fitting results, and the value R ranges as 0 < R < 1. Generally speaking, the closer the R-value is to 1, the closer the fitting result is to the expected result. The R values of the experimental training and test training results are 0.957 and 0.931, respectively, which shows that the ball landing point's initial velocity, rotation, and coordinates have a significant correlation.

**Figure 14:**
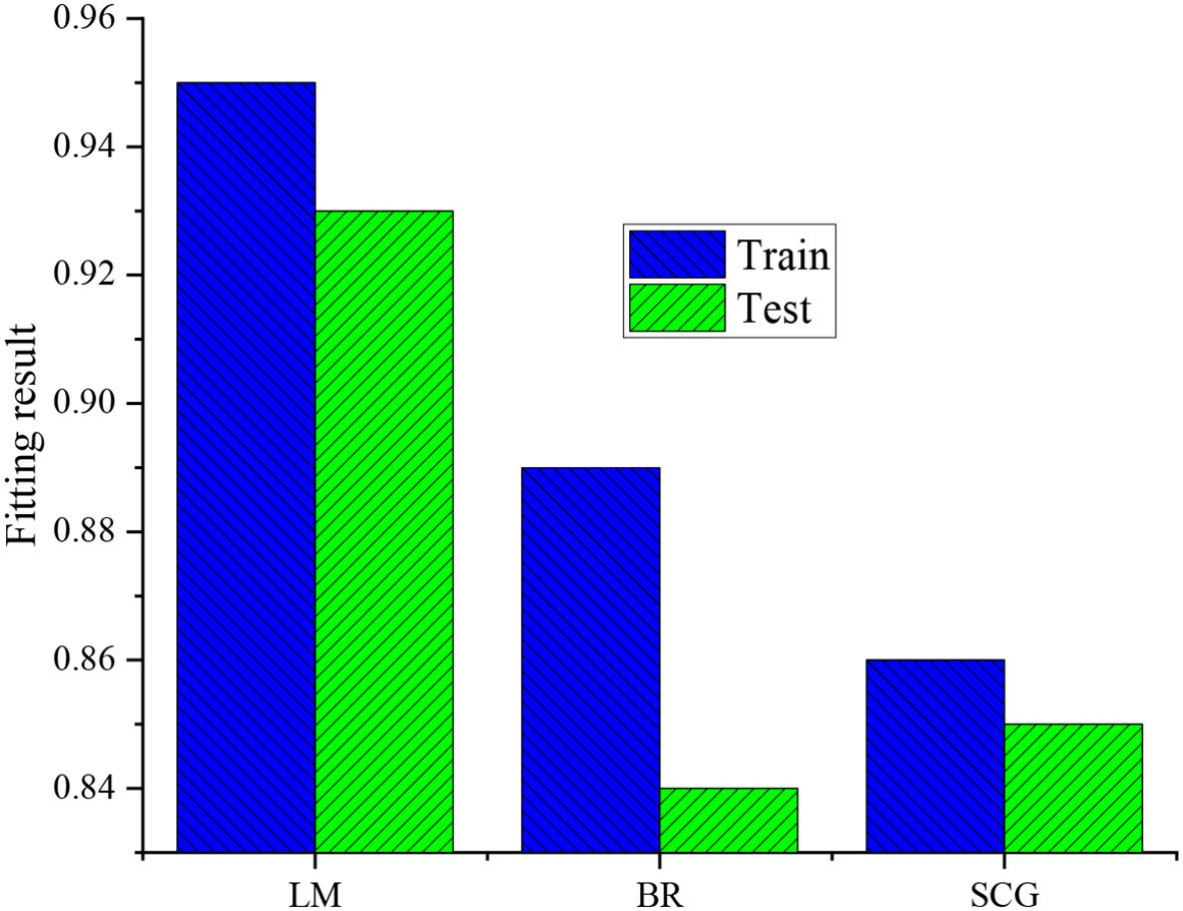
Fitting comparison results.

BR algorithm and SCG algorithm are used for comparison to verify the superiority of the LM algorithm. Figure 14 displays the comparison results.

Figure 14 illustrates that the fitting results of the LM algorithm are 0.95, while the fitting results of the BR algorithm and SCG algorithm are only 0.89 and 0.86. According to the test results, the *R*-value of the LM algorithm reaches 0.93, while the R values of the BR algorithm and SCG algorithm are only 0.84 and 0.85. Thus, no matter the training results or tests, the fitting condition of the LM algorithm is better than the BR algorithm and SCG algorithm. From the overall fitting situation, the fitting results of the three methods all exceeded 0.84. Therefore, even if different fitting algorithms are chosen, the CC between the expected and fitting results is very high, further showing that initial velocity, rotation, and coordinates of ball landing point have a solid correlation.

### Experimental Results of TTB Detection Based on FFN

**Figure 15:**
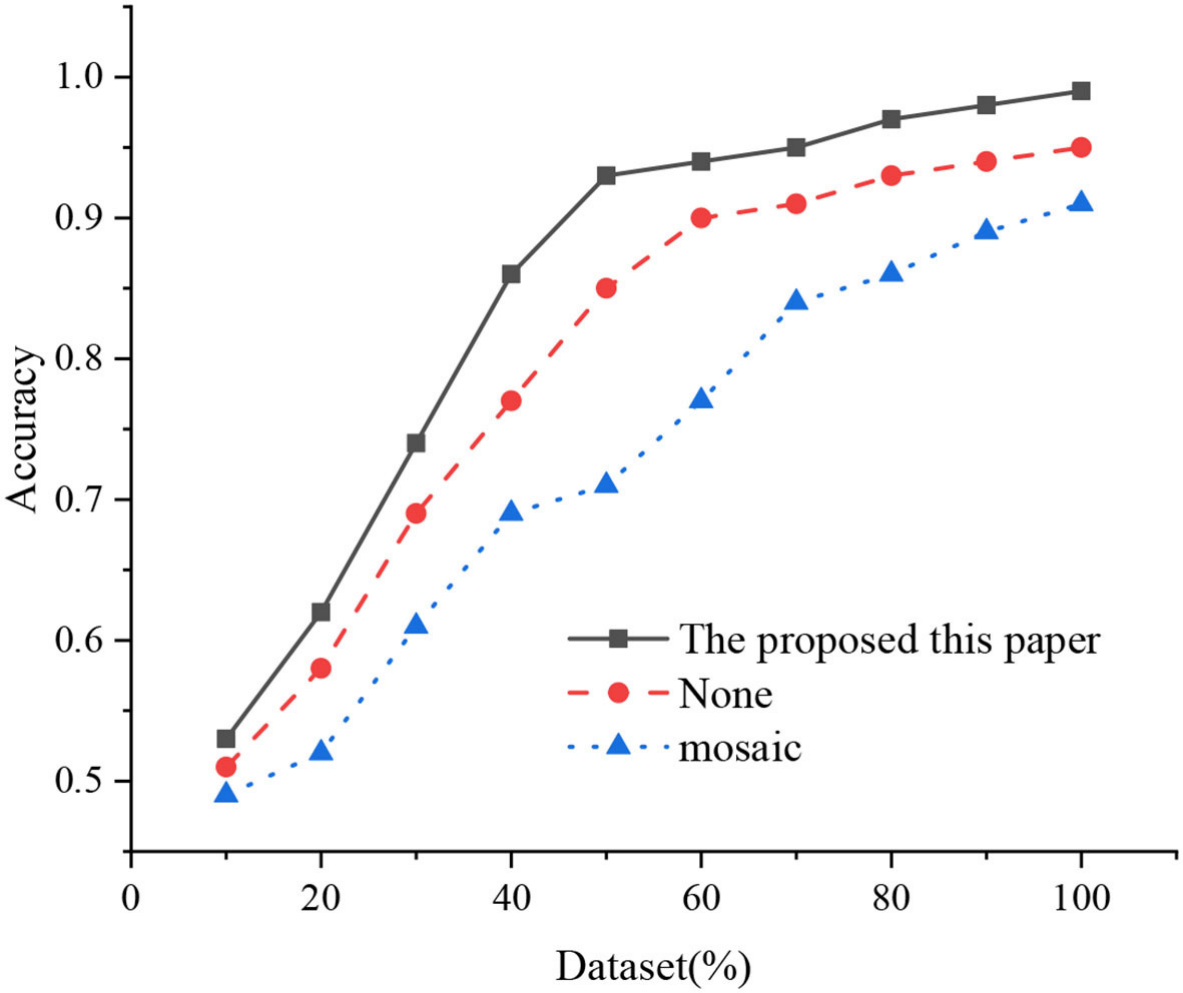
Accuracy comparison after data enhancement.

Subsequently, the experiment first uses Mosaic for data enhancement. Four images are spliced into one picture during network training and sent to network training. However, the detection effect of TTB is reduced rather than being improved as expected. The reason is probably that the size of TTB is scaled down after splicing, thus becoming more difficult for the network to obtain the TTB location and the decline of positioning accuracy. The TTB in each image is expanded three times without changing the image resolution and diversified training samples inspired by Mosaic enhancement and combined with the target features and subject requirements of TTB. Figure 15 illustrates the accuracy comparison after data enhancement.

**Figure 16:**
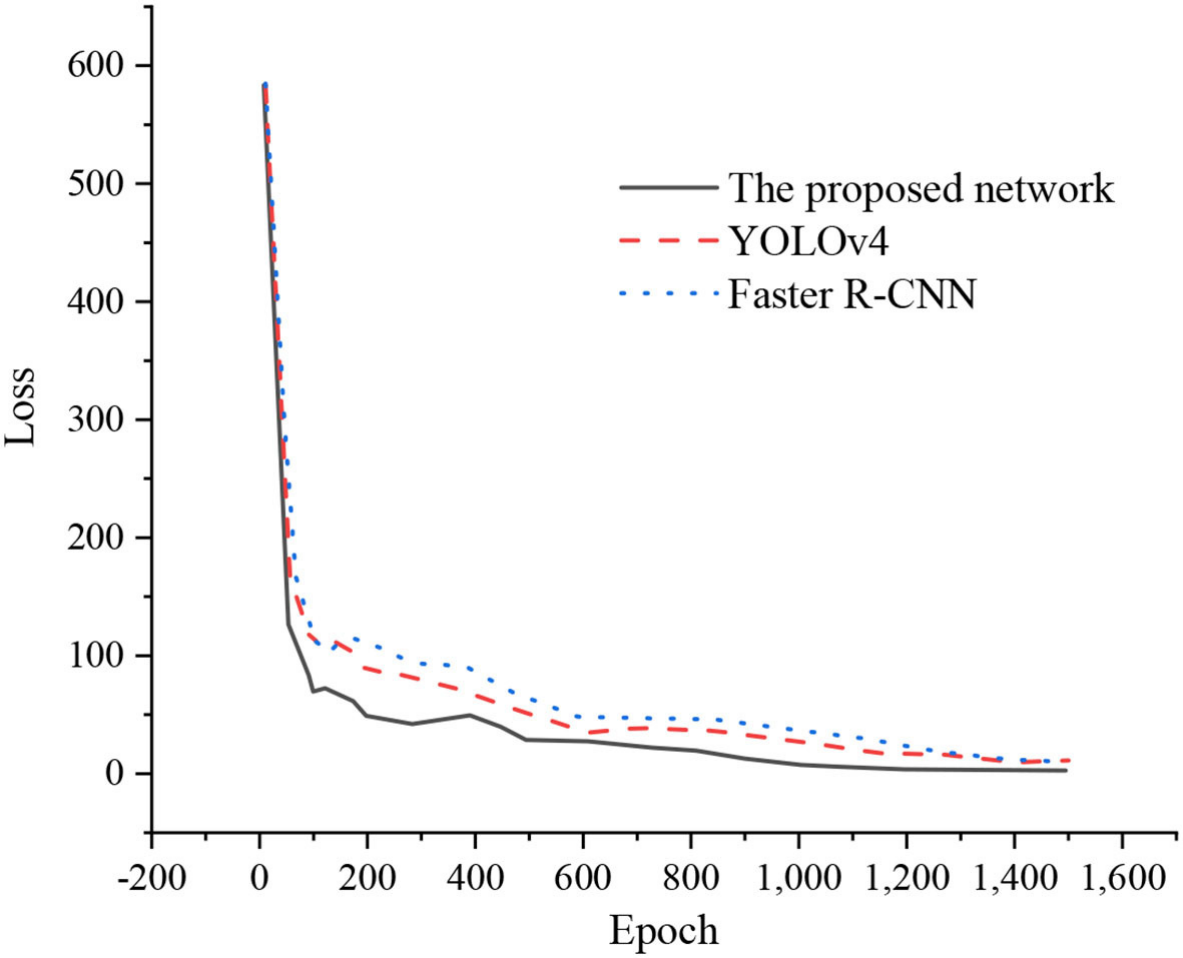
Network loss comparison.

This section builds a TTB-oriented target detection network using the Pytorch framework and Cuda to accelerate training. The dynamic calculation chart adopted by Pytorch can be changed and adjusted in real-time according to the actual calculation. At the same time, Pytorch also provides a toolkit for building a DL network with a clear and concise structure. It is a practical and efficient learning framework. In more than 8,000 data sets, this paper selects 7,000 images as the training set and the other 1,000 images as the test set. Then, Figure 16 compares the network losses of the training and test sets.

**Figure 17:**
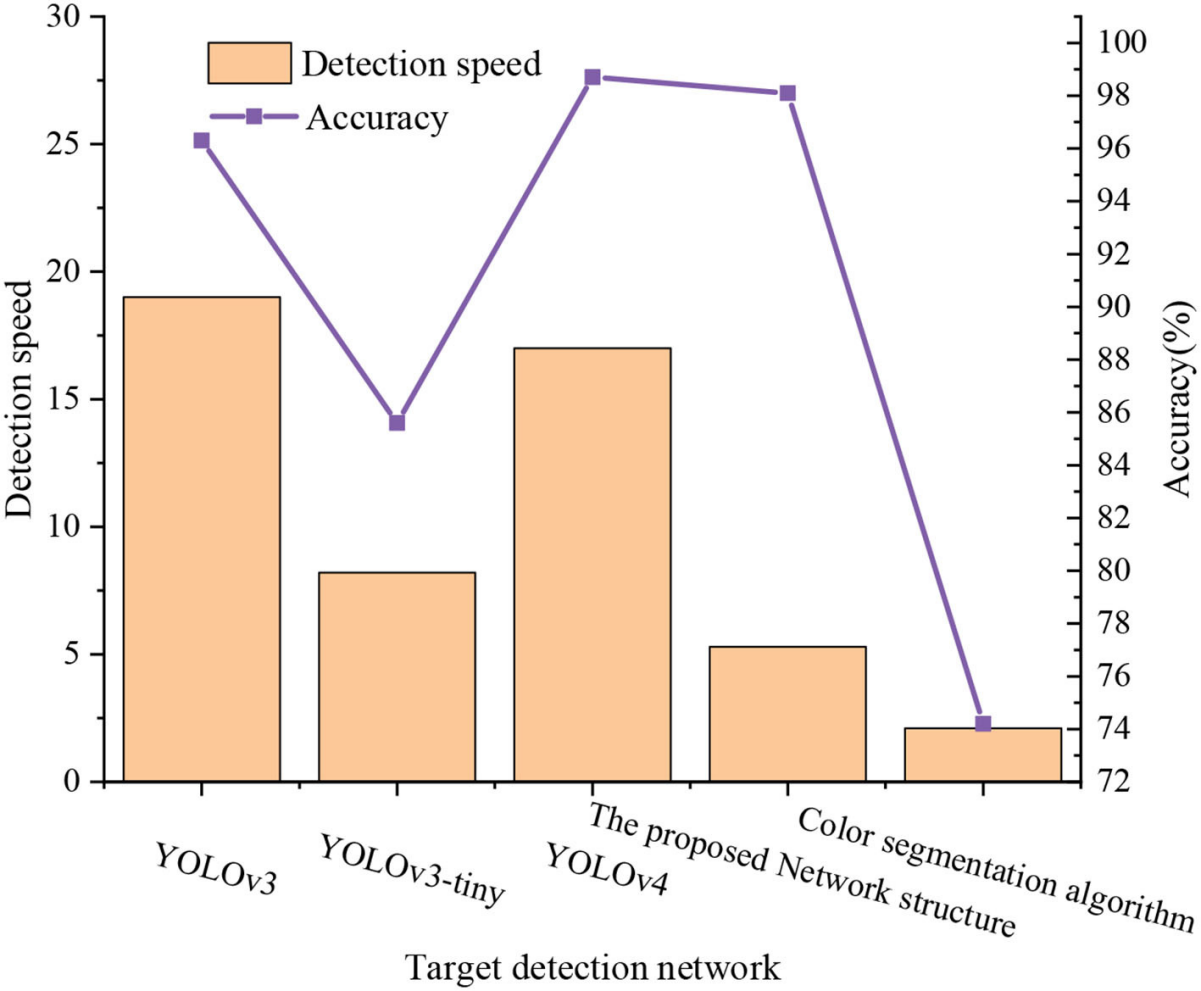
Experimental results comparison.

After training, the network detection accuracy can reach over 98%, and the detection response can be down to 5.3 ms, thus meeting the requirements of the table tennis vision system. First, the experiment compares some of the latest one-stage target detection networks, YOLOv3, YOLOv3-tiny, and YOLOv4, using the same laboratory equipment and the same training data set and test set. Figure 17 unveils the experimental results.

Figure 17 suggests that although the latest one-stage Yolo series models have achieved high accuracy in the TTB-oriented target detection task, the detection response is insufficient. Their network depth is more profound, and the amount of calculation and parameters are also more considerable, so they are not suitable for the research task of this study. The traditional color segmentation algorithm has advantages in detection response and can detect the target quickly, but the detection accuracy is not ideal. In particular, when the color of TTB changes, the detection ability will decline again. Therefore, the proposed TTB-oriented target detection network meets the table tennis real-time hitting and has very high accuracy.

### Experimental Results of the 7-DOF Table Tennis Robot System

Based on the physical system, this study has carried out the hitting experiments of different rotating balls, including the ball without spin, topspin, backspin, left-sidespin, and right-sidespin. The ball speed is slow (4 m/s) and fast (6 m/s). Table 2 provides the experimental results to analyze the feasibility and accuracy of this method in the physical system of TTR.

**Table 2:** Hitting test results.

	**Low speed**					**Fast speed**				
**Type**	**0**	**1**	**2**	**3**	**4**	**0**	**1**	**2**	**3**	**4**
Number of trails	200	50	50	50	50	200	50	50	50	50
A success times	97	–	–	–	–	69	–	–	–	–
A success rate/%	48.5	–	–	–	–	34.5	–	–	–	–
B success times	158	36	34	37	29	137	24	21	24	20
B success rate/%	79	72	68	74	58	68.5	48	42	48	40

In Table 2, A represents the comparative experiment, 0 represents the ball without spin, 1 denotes topspin, 2 signifies left-sidespin, 3 represents backspin, and 4 represents right-sidespin. In the case of a fast ball (6 m/s), the success rate of hitting the ball without spin can reach 68.5%. It can still ensure a specific success rate in receiving slow table tennis with spin. The results prove that the rotating ball discrimination method based on human experience reported here can take into account the spin characteristics of table tennis and guarantee the hitting attitude choice of the manipulator. However, the success rate of hitting the fast ball significantly decreases. Considering that the physical system of the seven-DOF KUKA mechanical arm used here has a slow control speed, the control system can only control the robotic arm to hit the ball through the slow mode. Consequently, the mechanical arm can only make effective hitting action after fast balls fly away from the table. Therefore, it is essential to improve the success rate of all kinds of balls in the rapid mode of a mechanical arm.

## Conclusion

This study mainly calculates and predicts the rotation, trajectory, and velocity of TTB using several AI algorithms. Experiments have proved a close correlation among speed, spin, and landing point of TTB, which provides a solid foundation for the reverse rotation of the TTB trajectory. Specifically, the experiment compares the kernel function difference of several Machine Learning algorithms on the model prediction performance. Finally, the optimal fitting method and kernel function is determined. Additionally, the algorithm based on Kinect depth camera plans the player's hitting action and predicts the rotation and trajectory of TTB by identifying and classifying the dynamic state of TTB. The results demonstrate that Kinect and SVM algorithms can achieve satisfying recognition results and high recognition accuracy in TTB-oriented target recognition, trajectory prediction, and rotation prediction. The network structure reported here has an excellent performance in predicting TTB motion state and player motion recognition.

Still, there are some challenges and problems in the process. First, most experiments are still in the laboratory environment. Follow-up research will focus on live games and use camera videos to enhance the recognition accuracy of TTB rotation. Second, there are many interference factors in real competition applications. Solving the interference of external factors is a crucial problem to be solved in future research. Third, the experiment uses insufficient samples and a single experimental environment. Thus, it is necessary to add more examples from other environments to calculate the rotation more accurately and improve the recognition accuracy of TTB.

## Data Availability Statement

The raw data supporting the conclusions of this article will be made available by the authors, without undue reservation.

## Ethics Statement

The studies involving human participants were reviewed and approved by Shanghai Polytechnic University Ethics Committee. The patients/participants provided their written informed consent to participate in this study. Written informed consent was obtained from the individual(s) for the publication of any potentially identifiable images or data included in this article.

## Author Contributions

All authors listed have made a substantial, direct, and intellectual contribution to the work and approved it for publication.

## Conflict of Interest

The authors declare that the research was conducted in the absence of any commercial or financial relationships that could be construed as a potential conflict of interest.

## Publisher's Note

All claims expressed in this article are solely those of the authors and do not necessarily represent those of their affiliated organizations, or those of the publisher, the editors and the reviewers. Any product that may be evaluated in this article, or claim that may be made by its manufacturer, is not guaranteed or endorsed by the publisher.
